# Radiosensitization by 2-benzoyl-3-phenyl-6,7-dichloroquinoxaline 1,4-dioxide under oxia and hypoxia in human colon cancer cells

**DOI:** 10.1186/1748-717X-2-1

**Published:** 2007-01-03

**Authors:** Wafica Itani, Fady Geara, Joelle Haykal, Makhluf Haddadin, Hala Gali-Muhtasib

**Affiliations:** 1 Department of Biology, American University of Beirut, Beirut, Lebanon; 2 Department of Radiation Oncology, American University of Beirut, Beirut, Lebanon; 3 Department of Chemistry, American University of Beirut, Beirut, Lebanon

## Abstract

**Background:**

The sensitizing effects of 2-benzoyl-3-phenyl-6,7-dichloroquinoxaline 1,4-dioxide (DCQ) and ionizing radiation (IR) were determined in four colon cancer cells and in FHs74Int normal intestinal cells.

**Methods:**

Cell cycle modulation, TUNEL assay, clonogenic survival and DNA damage were examined under oxia or hypoxia. Effects on apoptotic molecules and on p-Akt and Cox-2 protein expression were investigated.

**Results:**

The four cell lines responded differently to DCQ+IR; HT-29 cells were most resistant. Combination treatment caused significant increases in preG_1 _(apoptosis) in HCT-116, while G_2_/M arrest occurred in DLD-1. DCQ potentiated IR effects more so under hypoxia than oxia. Pre-exposure of DLD-1 to hypoxia induced 30% apoptosis, and G_2_/M arrest in oxia. The survival rate was 50% lower in DCQ+IR than DCQ alone and this rate further decreased under hypoxia. FHs74Int normal intestinal cells were more resistant to DCQ+IR than cancer cells.Greater ssDNA damage occurred in DLD-1 exposed to DCQ+IR under hypoxia than oxia. In oxia, p-Akt protein expression increased upon IR exposure and drug pre-treatment inhibited this increase. In contrast, in hypoxia, exposure to IR reduced p-Akt protein and DCQ restored its expression to the untreated control. Apoptosis induced in hypoxic DLD-1 cells was independent of p53-p21 modulation but was associated with an increase in Bax/Bcl-2 ratio and the inhibition of the Cox-2 protein.

**Conclusion:**

DCQ is a hypoxic cell radiosensitizer in DLD-1 human colon cancer cells.

## Background

Oxygen is known to help in stabilizing the radiation-induced DNA damage [1]. The lack of oxygen in solid malignant tumors results in their resistance to radiation therapy [1,2]. Attempts to overcome this resistance include the use of "oxygen-mimetic" radiosensitizers [3]; compounds which offer an attractive alternative for increasing the therapeutic window [4].

Quinoxaline 1,4-dioxides (QdNOs) share the di-*N*-oxide moiety with the clinically used drug Tirapazamine. These hypoxia-selective compounds are known to be redox-activated DNA-cleaving agents [5]. DNA cleavage by QdNOs requires enzymatic one-electron reduction of the compound to an activated, oxygen-sensitive intermediate [6]. This one-electron reduction is more likely to occur in the reducing conditions of hypoxic cells, targeting the toxicity of these compounds to hypoxic cells. Recent studies have shown that the nature of the substituent on the benzo-ring of the QDNO influences its potency [7]. Mild electron withdrawing groups in the 6(7) position increase the potency of these compounds under hypoxic conditions [7].

We have shown that the compound, 2-benzoyl-3-phenyl-6,7-dichloroquinoxaline 1,4-dioxide (DCQ), is a hypoxic cytotoxin [8]. Treatment of human colon cancer T-84 cells with DCQ reduced the expression levels of HIF-1α mRNA and protein [8]. The decrease in HIF-1α mRNA and protein expression by DCQ was later documented in EMT6 mouse mammary adenocarcinoma and Lewis lung carcinoma cells [9]. DCQ was also shown to reduce the expression levels of vascular endothelial growth factor (VEGF) and to inhibit hypoxia-induced angiogenesis [9]. Subsequent experiments performed by our group established that DCQ is an effective radiosensitizer both *in vitro *and *in vivo *[9]. When DCQ was combined with radiation, doses of 2.5–5 μM resulted in a dramatic decrease in clonogenic survival of EMT6 cells. The mechanism of radiosenitization by DCQ in EMT6 cells was found to involve the induction of G_2_/M arrest and apoptosis (unpublished results). Radiosensitization effects were also seen *in vivo *when LLC tumors were injected into C57BL/J6 mice and the effects of DCQ+IR on tumor volume were observed over 20 days [9].

This study aims, for the first time, to determine DCQ radiosensitizing activities in several human colorectal cancer cell lines and to investigate its cell cycle modulatory effects under both oxic and hypoxic conditions. Drug sensitization was examined in the FHs74Int normal human intestinal cell line to determine the sensitivity of normal cells to DCQ. In addition, the DNA damaging potential of DCQ and its effects on the protein expression levels of the oncogene Akt and on key molecules of apoptosis was investigated.

## Methods

### Cell culture

FHs74Int normal human intestinal cells were cultured in Hybri-Care medium supplemented with 30 ng/ml epidermal growth factor. Human colon cancer cell lines (DLD-1, HT-29, HCT-116, and SW-480) were grown in RPMI 1640 containing L-Glutamine and 25 mm HEPES. All media were supplemented with 10% heat-inactivated FBS and 1% Penicillin-Streptomycin (50 μg/ml). Cells were cultured in a humidified incubator (95% air 5% CO_2_) at 37°C (Forma Scientific Inc. Ohio, USA).

### Drug preparation

DCQ was synthesized from 5,6-dichlorobenzofurazan oxide and dibenzoylmethane according to the Beirut Reaction [10]. A fresh stock of 10 mg of DCQ was dissolved in 1 ml of filtered DMSO. Before treatment, DCQ was diluted 1 in 10 using media containing 10% FBS and 1% Penicillin-Streptomycin (50 μg/ml).

### Radiation experiments

Cells cultured in 25 cm^2 ^T-flasks were treated either with DCQ (0–10 μM), irradiation (0–6 Gy) or combinations. Irradiation was administered by a JL Shepherd, 143-68 Cesium-137 Laboratory Irradiator with an output activity of 1683 Ci. Immediately after irradiation, cells were replenished with fresh media containing no drugs and left in the incubator for 24 hours for studies on cell cycle regulation and DNA damage (COMET) as described below.

### Hypoxia treatment

DLD-1 or FHs 74Int cells cultured in 25 cm^2 ^flasks were treated at 50% confluency with DCQ for 4 hours, after which they were placed in a tightly sealed chamber (37°C, 1% O_2_) for 1 hour. The desired oxygen level was optimized by injecting N_2 _gas into the chamber, and the levels were measured every 15 minutes using an Ohmeda Oxymeter (Datex-Ohmeda, Louisville, CO). Immediately after hypoxia the flasks were sealed and the cells were irradiated. Later, cells were replenished with fresh media containing no drugs and incubated for another 24 hours.

### Clonogenic survival

Oxic or hypoxic DLD-1 cells cultured in 25 cm^2 ^T-flasks were treated with DCQ (0–100 μM, 1 hour), after which they were irradiated (2 Gy). FHs74Int cells were treated under oxic conditions with DCQ (0–10 μM) for 1 hour prior to irradiation (2 Gy). Immediately after irradiation, both cell lines were re-plated at known dilutions with fresh media for 10 days. After 10 days of incubation, colonies were stained with crystal violet and counted. The number of colonies containing more than 50 cells was counted and the percentage of survival rates at each dose was calculated according to the formula: (colony no. in treatment/colony no. in control) × 100.

### Cell cycle analysis using flow cytometry

Following treatment, cells were harvested, fixed in ice cold 70% ethanol and stored at -20°C. On the day of DNA staining, cells were incubated for 75 minutes in 200 μg/ml RNase A at 37°C, and stained with 50 μg/ml propidium iodide. Cell cycle analysis was performed using a FACScan flow cytometry (Becton Dickinson, Research Triangle, NC) and the percentage of cells in preG_1_, G_1_, S, and G_2_/M phases was determined using the Cell Quest program.

### Apoptosis TUNEL assay

Fragmented DNA was detected by Terminal deoxy-transferase (TdT)-mediated dUTP nick-end labeling (TUNEL assay) (Roche Diagnostics, Mannheim, Germany) to assess the induction of apoptosis. Following treatment, cells were harvested and the pellet was suspended in 100 μl freshly prepared PBS in 4% formaldehyde, incubated at room temperature for 30 minutes, and centrifuged at 300 g/2000 rpm for 10 minutes. The pellet was washed once with 200 μl PBS. Followed by suspension in 100 μl of a solution containing 1× PBS, 0.1% sodium citrate, and 0.1% Triton X-100 for 2 minutes on ice. Cells were then washed twice with 1× PBS. The pellet was resuspended in 50 μl tunnel reaction mixture (45 μl labeling solution and 5 μl enzyme solution), incubated for 1 h at 37°C in a humidified atmosphere in the dark, then washed twice with 1× PBS and suspended in 1× PBS for reading by flow cytometry. Cells suspended in 50 μl labeling solution served as the negative control. The samples were examined by FACScan flow cytometer to determine the percentage of apoptotic cells in treated samples as compared to the control samples.

### Single Cell Gel Electrophoresis (SCGE)/comet assay

DNA damage, including single strand breaks (SSB) and alkali labile sites (ALS), was measured using the alkaline SCGE assay in DLD-1 cells treated with DCQ (5 μM, 1 hour) IR (2 Gy) or combinations under oxia or hypoxia. Immediately after IR, cells were scraped and collected in RPMI medium. Comet assay was performed as described previously [11]. For electrophoresis, an electric current of 25 volts and 300 mA was applied for 30 minutes, after which the slides were placed in a neutralizing buffer for 5 minutes. This neutralizing procedure was repeated two more times. Finally, 50 μl of YOYO stain (0.25 μM YOYO, 2.5% DMSO and 0.5% sucrose) (Molecular Probes – Eugene, Oregon, USA) was added to each slide and analyzed immediately using a fluorescence microscope (AXIOVERT 200, ZEISS Flourescence and optical microscope with ZEISS AXIOCAM HRC and KS 300 V3 image analysis software). Images of 100 randomly selected non-overlapping cells (magnification 100×) were analyzed for each sample with the help of Tri-Tek CometScore™ software, a fully automatic image analysis system. The following parameters were used to assess DNA damage: total fluorescence of the comet, fluorescence of the tail, percentage of DNA in the tail region and tail moment (%DNA in tail multiplied by tail length). The comet data values were expressed as mean ± S.D. Statistical comparisons were made by *t*-test and the *P*-values < 0.05 or *P *< 0.01 were considered significant.

### Protein expression by Western Blotting

DLD-1 cells cultured in 75 cm^2 ^T-flasks were treated with DCQ (5 μM, 1 hour), IR (2 Gy) or combinations under oxic or hypoxic conditions. Cellular proteins were extracted by SDS-lysis buffer (50 mM Tris-HCL, pH 7.5, 150 mM NaCl, 1% Nonidet P40, 0.5% Sodium deoxycholate, 4% protease inhibitors and 1% phosphatase inhibitors). Protein extracts were centrifuged for 10 minutes at 14,000 rpm. Proteins were quantified using the DC Bio-Rad Protein Assay kit with BSA as a standard. Whole cell lysates (40–60 μg) were loaded on 12% SDS-polyacrylamide gels and then transferred onto PVDF membranes (Amersham Pharmacia Biotech, Amersham, England). The membranes were incubated with the primary antibodies: p21 (F-5), p53 (FL-393), p-p53, Bcl-2 (N-19), Cox-2 (all from Santa Cruz, CA), Bax (Biosource, California, USA), pS473 Akt (44-622G) (Chemicon International, California, USA). The GAPDH antibody (Biogenesis, Poole, UK) was used as a loading control. The membrane was then washed 3 times for 10 minutes each in wash buffer (TBS containing 0.05%–0.1% Tween 20) and probed with the appropriate secondary antibody (IgG-HRP, antirabbit IgG-HRP, or antigoat IgG-HRP from Santa Cruz) for 1 hour at room temperature. After wash, the membrane was exposed to X-ray film (Hyperfilm ECL, Lebanon) using a chemiluminescent substrate (Amersham Pharmacia Biotech, Amersham, England). The bands were quantified using LabWorks 4.0 software.

## Results

### Cell cycle modulation in four human colon cancer cell lines under oxia

**Figure 1:**
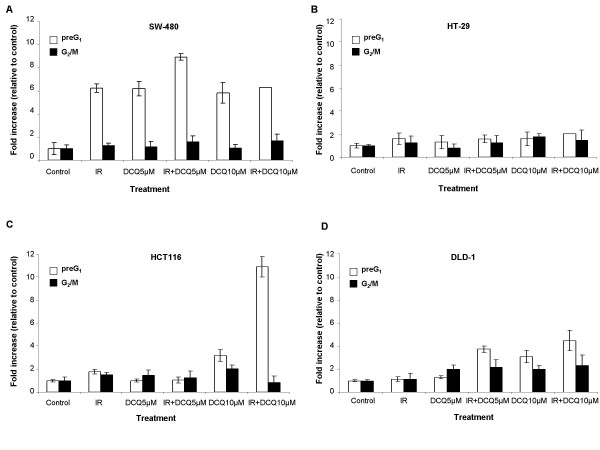
Effect of DCQ, IR and their combinations on cell cycle regulation in four different human colon cancer cell lines (SW-480, HT-29, HCT116 and DLD-1). Cells were treated with DCQ (0, 5, 10 μM), IR (2 Gy) or combinations. Immediately after radiation or drug treatment, cells were replenished with fresh medium containing no drug and incubated for another 24 hours. Control cells were treated with DMSO (0.1%). Cell cycle changes were assessed using Propidium Iodide stain with flow cytometry as described in "Materials and Methods". The percentage of cells in preG_1 _and G_2_/M phases were plotted as a function of DCQ dose. Results are representative of at least two independent experiments each performed in duplicates.

To study cell cycle modulation by DCQ+IR, cells were incubated with DCQ (5 or 10 μM) for either 1 hour (DLD-1 and HCT116) or 4 hours (SW-480 and HT-29), and then irradiated (2 Gy). The times, 1 or 4 hours, were chosen based on differences in the sensitivity of the four cell lines to the drug. While SW-480 and HT-29 survived after 4 hour exposure to DCQ, DLD-1 and HCT-116 died when drug treatment was extended for more than 1 hour (data not shown). Twenty four hours after treatment, cells were harvested for flow cytometry analysis and the percentage of cells in preG_1 _and G_2_/M phases were plotted as these phases were the most modulated. The response of the four cell lines to DCQ+IR was different; HT-29 cells were the most resistant followed by SW-480 (Figure 1A and 1B). HCT116 and DLD-1 were sensitive to DCQ+IR, but responded differently. Treatment with 10 μM DCQ+IR caused 11 fold increases in the preG_1 _portion in HCT-116 (Figure 1C), however, in DLD-1 cells 2-fold increases in the percentage of G_2_/M cells was observed (Figure 1D).

### Cell cycle modulation in HCT116 and SW-480 cells under hypoxia

**Figure 2:**
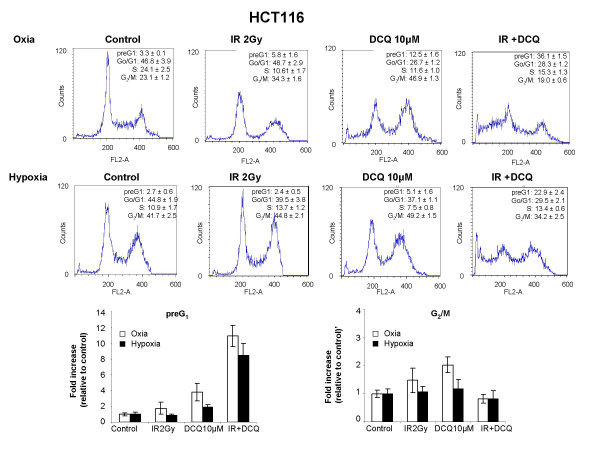
Effect of DCQ, IR and their combinations on cell cycle regulation in HCT116 cells exposed to oxic or hypoxic conditions. Cells were treated with 5 μM DCQ or DMSO (0.1%) and exposed to hypoxia or incubated in oxia for 1 hour, then irradiated (2 Gy). Immediately after radiation or drug treatment, cells were replenished with fresh medium containing no drug and incubated for another 24 hours. Cell cycle changes were assessed using Propidium iodide stain with flow cytometry as described in "Materials and Methods". Bar graphs are a summary of at least three independent experiments each performed in duplicates.

**Figure 3:**
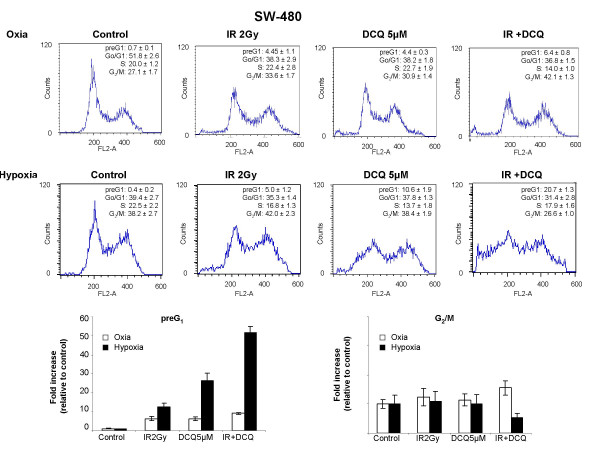
Effect of DCQ, IR and their combinations on cell cycle regulation in SW-480 cells exposed to oxic or hypoxic conditions. Cells were treated with 5 μM DCQ or DMSO (0.1%) and exposed to hypoxia or incubated in oxia for 4 hours, then irradiated (2 Gy). Immediately after radiation or drug treatment, cells were replenished with fresh medium containing no drug and incubated for another 24 hours. Cell cycle changes were assessed using Propidium iodide stain with flow cytometry as described in "Materials and Methods". Bar graphs are a summary of at least three independent experiments each performed in duplicates.

Since DCQ is a hypoxic cytotoxin [9], we then investigated whether it could potentiate IR effects more so under hypoxia than oxia. The hypoxia toxicity of DCQ was first studied in the two cell lines, HCT116 and SW-480. Cells were incubated in DCQ (5 μM, 1 or 4 hours) under oxic or hypoxic conditions, after which they were irradiated, then replenished with media containing no DCQ, and harvested 24 hours later for cell cycle analysis (Figures 2 and 3). In both cell lines, hypoxia treatment alone caused G_2_/M arrest (1.5–2.0 fold increase). Exposure of HCT-116 cells to oxic or hypoxic conditions prior to IR resulted in no difference in their sensitivity to the drug (% of cells in preG_1 _phase was 36% in oxia and 23% in hypoxia) (Figure 2). However, SW-480 showed a significant increase in preG_1 _cells when combination treatment was done under hypoxia (Figure 3). Considering that HCT116 and SW-480 were sensitive to hypoxia, no further studies were done with these cell lines.

### Cell cycle modulation and clonogenic survival in DLD-1 cells under hypoxia

**Figure 4:**
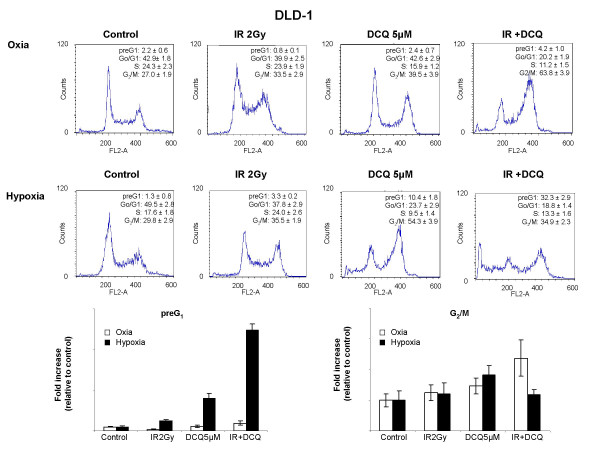
Combination effects of DCQ and IR in DLD-1 cells under oxic and hypoxic conditions. Cells were treated with 5 μM DCQ or DMSO (0.1%) and exposed to hypoxia or incubated in oxia for 1 hour, then irradiated (2 Gy). Immediately after radiation or drug treatment, cells were replenished with fresh medium containing no drug and incubated for another 24 hours. Cell cycle changes were assessed using Propidium iodide stain with flow cytometry as described in "Materials and Methods". Bar graphs are a summary of at least three independent experiments each performed in duplicates.

To investigate the hypoxic cytotoxicity of DCQ in DLD-1, we compared its efficacy in cells incubated in oxia or hypoxia prior to irradiation. DLD-1 cells were treated with DCQ (5 μM) + IR and harvested after 24 hours for cell cycle analysis (Figure 4). Treatment under oxia resulted in the accumulation of 63% of the cells in G_2_/M phase and 4% in preG_1_. More pronounced effects were observed in hypoxia, as 33% of apoptotic cells accumulated in preG_1 _(Figure 4). Therefore treatment of DLD-1 cells with DCQ+IR caused G_2_/M arrest in oxia and preG1 arrest in hypoxia.

**Figure 5:**
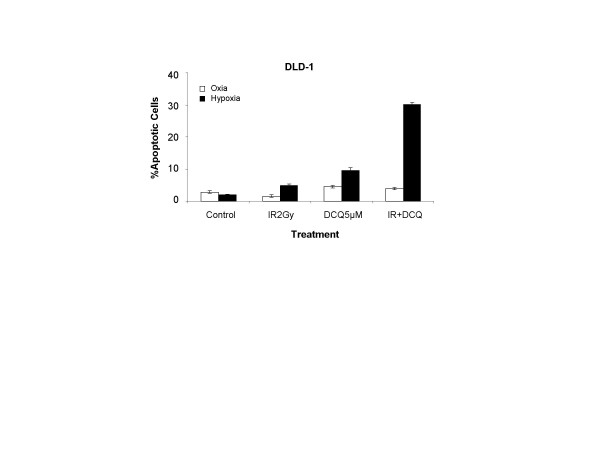
TUNEL assay showing that the combination of DCQ and IR induces apoptosis in DLD-1 cells under oxic and hypoxic conditions. Cells were treated with 5 μM DCQ or DMSO (0.1%) and exposed to hypoxia or incubated in oxia for 1 hour, then irradiated (2 Gy). Immediately after radiation or drug treatment, cells were replenished with fresh media containing no drugs and left in the incubator for 24 hours. The extent of DNA fragmentation was determined by TUNEL assay and measured by flow cytometry. The percentage of apoptotic cells was determined using CellQuest. Results are representative of at least two independent experiments.

Using TUNEL assay, the level of apoptosis in cells treated with DCQ+IR under oxic and hypoxic conditions was found to be 3.9% and 30% respectively (Figure 5) confirming that the increases in preG1 observed by flow cytometry are due to apoptosis.

**Figure 6:**
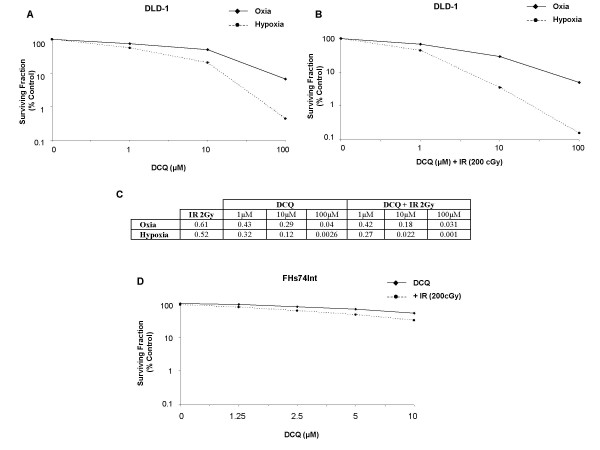
Survival curves of DLD-1 cancer cells and FHs74Int normal cells exposed to DCQ alone or DCQ and irradiation. A. DLD-1 cells were exposed to 1 hour oxia or hypoxia in the presence of DCQ and the surviving fraction was determined as a percentage with respect to the untreated cells. B. DLD-1 cells were exposed to 1 hour oxia or hypoxia in the presence of DCQ and then irradiated (2 Gy) and the surviving fraction was determined as a percentage with respect to the irradiated cells. C. Absolute survival rates of DLD-1 cells exposed to DCQ, IR or their combinations under oxic and hypoxic conditions. D. FHs74Int cells were exposed to 1 hour oxia in the presence of DCQ and then irradiated. After irradiation, cells were re-plated and the colonies were stained with crystal violet and counted 10 days later. Each data point was calculated as percent of untreated cells of two independent experiments each performed in duplicates.

To confirm the hypoxic effects of DCQ, DLD-1 cells were treated with DCQ (1–100 μM) in oxia or hypoxia, irradiated (2 Gy) and then re-plated at known dilutions. Ten days after re-plating, the surviving colonies were counted. The survival curves for DCQ+IR and DCQ alone show a more pronounced decrease in cell survival under hypoxia than oxia (Figure 6). Exposing DLD-1 cells to IR alone did not reduce the absolute survival rate of cells under hypoxia as compared to oxia (Figure 6C). When DLD-1 cells were exposed to DCQ alone (10 μM), the surviving fraction determined with respect to the untreated cells was 0.49 (SD ± 0.04) in oxia and 0.20 (SD ± 0.02) in hypoxia (Figure 6A). However, when DCQ (10 μM) was combined with IR, the surviving fraction determined with respect to the irradiated cells dropped to 0.29 (SD ± 0.03) in oxia and 0.04 (SD ± 0.01) in hypoxia (Figure 6B).

The hypoxia cytotoxicity ratio (HCR), i.e. the concentration of drug required under oxia relative to hypoxia to produce 90% cell death, was 4 fold higher when DCQ was combined with IR (HCR = 12) as compared to DCQ alone (HCR = 3). This provided additional evidence that the drug is a potent radio-sensitizer in hypoxic cells.

### DCQ radiosensitization in the FHs74Int normal intestinal cell line

After establishing effects of DCQ and IR in cancer cells, we compared DCQ efficacy in normal cells. For this purpose, FHs74Int normal human intestinal cells were pre-treated with DCQ (1.25–10 μM, 1 hour), irradiated, and then re-plated at known dilutions and the surviving colonies were determined 10 days later. At 5 μM DCQ, the survival rate was 0.68 (SD ± 0.02), and this rate was reduced to 0.46 (SD ± 0.01) when DCQ (5 μM) was combined with IR (Figure 6D). A comparison of the extent of decrease in cell survival in DCQ+IR in normal FHs74Int *v.s. *DLD-1 cancer cells confirms the greater radio-sensitizing effects of this drug in cancer cells.

### DNA damage by DCQ in irradiated DLD-1 cells under oxia and hypoxia

**Figure 7:**
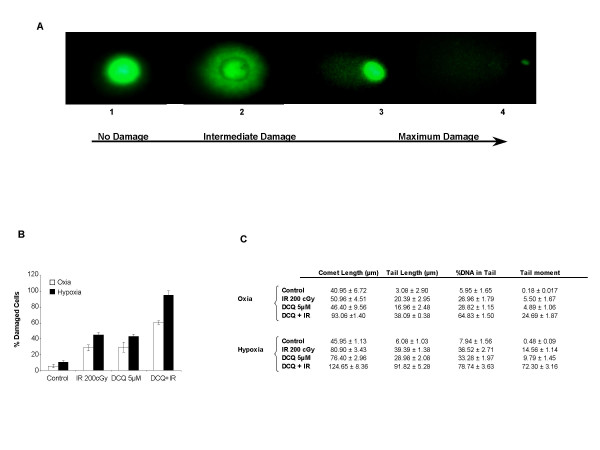
Induction of DNA damage in DLD-1 cells after treatment with DCQ, IR or combinations under oxic and hypoxic conditions. Cells were treated with 5 μM DCQ for 1 hour, 2 Gy IR or combinations. Immediately after treatment, DNA damage was assessed using alkaline single cell microgel electrophoresis (Comet) assay as mentioned in the "Materials and methods" section. A. The figure shows different grades of DNA fragmentation in DLD-1 cells. Magnification: 100×. B. An average of 100 cells per slide were counted and analyzed, and the mean of damaged cells is represented as the percentage of control untreated cells. C. Quantitative measurements of various comet assay end-points as analyzed using Comet Score software.

To determine if DCQ is a DNA-targeting agent, the extent of DNA damage was measured by the alkaline COMET assay in oxic or hypoxic DLD-1 cells exposed to DCQ (5 μM, 1 hour), IR or combinations. The COMET assay measures single strand DNA breaks by the increase in the electrophoretic mobility of denatured genomic DNA in an agarose gel. Figure 7A shows an example of different grades of DNA fragmentation. In the first image, the DNA of a largely non-fragmented cell is depicted. The next 2 images represent cells with increasingly fragmented DNA; thus giving the comet its tail. The last image shows a cell with highly fragmented DNA. Treatment with DCQ+IR resulted in a statistically significant increase (*p *< 0.01) in DNA damage in hypoxia compared to oxia. The mean percentage of DNA damage was 95 (SD ± 5.65) in cells exposed to DCQ+ IR under hypoxia as compared to only 60.5 (SD ± 2.12) under oxia (Figure 7B).

Digital images were further analyzed using Comet Score software that allows quantitative measurements of various comet assay end-points, in particular, the mean average of comet length, tail length, and percentage of DNA in the tail (Figure 7C). In addition, tail moment was calculated as the product of the percentage of DNA in the comet tail multiplied by the total comet length. Such end-points are the most accepted parameters for assessing DNA damage. It is important to note that 1 hour exposure of the cells to hypoxia did not induce a major change in any of the measured comet assay end-points.

Several end-point measures indicated that DCQ is a more potent DNA damaging agent in irradiated hypoxic cells: 1) significant (*p *< 0.05) increase in mean tail moment in hypoxia compared to oxia (24.69 in oxia *v.s. *72.3 in hypoxia); 2) greater relative amount of damage, quantified by measuring the distance that DNA moves in the gel or the length of the comet tail; 3) greater amount of DNA present in the tail in hypoxic cells (11 fold increase in tail DNA in hypoxia *v.s. *7-fold increase in oxia) (Figure 7C).

### DCQ effects on radiation-induced p53, p-p53 and p21 expression

**Figure 8:**
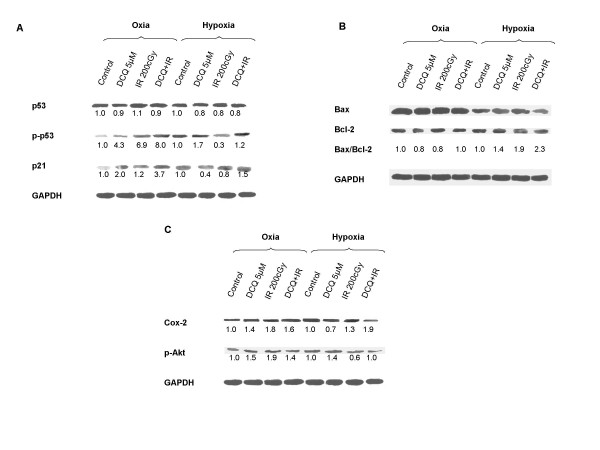
Effects of DCQ and IR on the expression levels of p53, p-p53, p21 (A), Bax/Bcl2 (B), p-Akt and Cox-2 (C) proteins. DLD-1 cells were treated under oxic and hypoxic conditions with 5 μM DCQ, 2 Gy IR or combinations. After 24 hours, 40 μg cell lysates were subjected to SDS-PAGE. Fold induction of protein levels was based on densitometry measurments. Protein levels in treated cells were defined as percentage of control. All plots were re-probed with GAPDH to ensure equal protein loading.

To investigate the effects of DCQ on key apoptotic molecules, DLD-1 cells were treated with DCQ, IR or combinations under oxic or hypoxic conditions and the expression levels of p53, p-p53 and p21 proteins were determined (Figure 8). The phosphorylation of p53 normally stabilizes the protein [12,13] which in turn activates and stabilizes p21 leading to cell cycle arrest [14,15]. In hypoxia, the IR-induced p53 protein expression levels were reduced by 0.3 fold in cells exposed to DCQ prior to IR (Figure 8A). A much greater increase in the expression levels of p-p53 protein was evident in cells exposed to DCQ+IR under oxia (8 fold) than hypoxia (1.3 fold) (Figure 8A). This increase was associated with an increase in p21 protein expression levels under oxia (3.7 fold) and hypoxia (1.5 fold) (Figure 8A). This finding aligns with the fact that the induction of p21 under hypoxia may be independent of p53 status.

### DCQ effects on radiation-induced Bax/Bcl-2 expression

We then investigated whether DCQ radiosensitization is associated with changes in the levels of the anti-apoptotic Bcl-2 and pro-apoptotic Bax proteins. Up regulation of Bax and down regulation of Bcl-2 favor the pro-apoptotic over the anti-apoptotic response in the cell leading to the release of cytochrome c and promoting cell death. Treatment with DCQ+IR in oxic cells did not induce changes in the Bax/Bcl-2 ratio (Figure 8B). However, DCQ+IR in hypoxic cells increased Bax/Bcl-2 expression by 2.3 fold.

### DCQ effects on radiation-induced p-Akt expression

Since the Akt survival oncogene is known to be involved in the transition to G_2_/M [16], its inhibition may lead to cell cycle arrest at G_2_/M phase. In oxic cells, p-Akt protein expression levels increased upon exposure to IR; pretreatment with DCQ inhibited this increase in p-Akt protein (Figure 8C). In contrast, in hypoxic cells, exposure to IR reduced p-Akt protein expression levels and DCQ restored those levels to the untreated control (Figure 8C). It appears that the inhibition of p-Akt by DCQ under oxia results in enhanced susceptibility of DLD-1 cells to IR, thus leading to cell cycle arrest at G_2_/M.

### DCQ effects on radiation-induced Cox-2 expression

Cox-2 is an anti-apoptotic protein the expression of which is reduced at high Bax/Bcl-2 protein expression levels [17]. Therefore, we examined whether DCQ radiosensitization is associated with changes in the Cox-2 protein (Figure 8). Recent studies show that Cox-2 inhibition can restore p53 levels in response to hypoxia and thereby render the cells more sensitive to therapeutic agents [18]. DLD-1 cells exposed to hypoxia had 1.7 fold higher levels of Cox-2 protein than those exposed to oxia (Figure 8C). Pre-treatment with DCQ was found to inhibit the IR-induced levels of Cox-2 protein by 0.2 fold in oxic cells and by 9.8 fold in hypoxic cells. It is interesting to note that the significant inhibition of Cox-2 protein by DCQ in hypoxic and irradiated cells is associated with increased p-p53 protein levels and Bax/Bcl-2 ratio (Figure 8C). Such protein modulation may be responsible for the greater DCQ radiosensitization in hypoxic cells.

## Discussion

The use of non-toxic drugs that are activated in hypoxic regions of tumors are known to enhance the killing effects of radiation therapy and to be the most effective treatment modality so far [19]. Here, we demonstrate that DCQ is a DNA-damaging radiosensitizer with greater efficacy towards hypoxic tumor cells. This is the first report of DCQ sensitization when combined with IR against human colon cancer cells.

All four human colon cancer cell lines were sensitive to DCQ+IR, but to a different extent. Although HT-29 cell line was resistant, the three other cell lines (HCT116, SW-480, DLD-1) showed relative sensitivity towards the combination of DCQ and radiation. The efficacy of the drug was enhanced when the cells were exposed to hypoxia prior to irradiation. The combination of drug and radiation treatment under hypoxia resulted in apoptosis, while such treatment induced G_2_/M arrest in oxic cells. This indicates that DCQ enhances IR effects to a different extent according to the cell type, and G_2_/M arrest and apoptosis are involved in the mechanism of radiosensitization by the drug. Interestingly, normal cells were less sensitive to DCQ sensitization than cancer cells.

Using the alkaline Comet assay, DCQ was found to be a redox-activated DNA-damaging agent when combined with radiation, with selective toxicity against hypoxic cells. Recent evidence indicates that the hypoxia selective cytotoxic activity of quinoxaline 1,4-dioxides involves enzymatic reduction of the compound to a crucial oxygen-sensitive radical intermediate capable of cleaving the DNA [7]. Many QdNOs are known as "chemical nucleases" that efficiently "nick" the DNA [20]. Most prominent among these compounds is 3-amino-1,2,4-benzotriazine1,4-dioxide (tirapazamine TPZ), a heterocyclic di-N-oxide that is selectively toxic to hypoxic tumor cells. TPZ is also involved in transferring oxygen atoms from its N-oxide functional groups to these radicals, converting them to base-labile strand cleavage sites [7].

A significant increase in DNA single strand breaks, measured as alkaline tail moment, was observed in DLD-1 cells exposed to DCQ and IR under hypoxic conditions. However, DCQ and IR under oxic conditions predominantly induced relatively non-cytotoxic single-strand breaks. DNA single strand breaks or alkali labile sites are by far the largest number of lesions in DNA in general. Therefore, the decrease in cell survival and induction of apoptosis in DLD-1 cells was likely due to the additive effects of DNA damage produced by DCQ and IR upon hypoxia. On the basis of structural correlation between TPZ and the quinoxaline 1,4-dioxide DCQ, the latter compound can be considered as a more potent DNA radical oxidant by oxidizing such DNA radicals to cytotoxic DNA strand break [3].

Studies have reported the influence of the cellular p53 status on radiosensitivity, due to the function of this tumor suppressor gene in the cellular response to DNA damage [21]. Activation of p53 following genotoxic damage is achieved by the induction of p53 levels and by the phosphorylation of the p53 protein, in particular, at serine 15 and 20 [22]. Here we show that irradiating hypoxic DLD-1 cells reduced the protein expression levels of p-p53, while DCQ in combination with IR caused no changes in p53 or p-p53 protein. This suggests that the enhanced response of hypoxic DLD-1 cells to the combination treatments is probably due to the radiation-induced reduction of p53 as a result of increased DNA instability at various loci [23]. However, p-p53 protein levels were increased in DLD-1 cells treated with DCQ and IR under oxic conditions, indicating that p53 may be involved in the mechanism by which DCQ and IR induce cell cycle arrest at G_2_/M phase; the most radiosensitive phase of the cell cycle.

The mechanism by which p53 induces cell-cycle arrest is highly dependent upon the transcriptional induction of p21, which inhibits cyclin dependent kinase activity that is necessary for G_2_/M transitions [24]. Our findings show that p53-p21 signaling pathways may be involved in DCQ radiosensitization under oxia but not under hypoxia in DLD-1 cells. This indicates that hypoxia enhances DCQ's potent activity as radiosensitizer through a different mechanistic pathway than what is observed under oxia.

It appears that the induction of cell death in hypoxic DLD-1 cells after combination treatments involves the induction of Bax/Bcl-2 expression levels. Among the variety of proteins that control the apoptotic program are the members of the Bcl-2 family that act as inhibitors (Bcl-2, Bcl-Xl and Bcl-W), and those that act as promoters of apoptosis (Bax, Bad, Bak and Bcl-Xs) [25]. We showed that hypoxia enhances the expression of Bcl-2 protein and reduces Bax protein expression levels, thereby inhibiting apoptosis. DNA damage could trigger apoptosis via a p53-mediated pathway that includes the upregulation of the pro-apoptotic protein Bax [26]. In our study, treatment with DCQ plus radiation under hypoxic conditions in DLD-1 cells down regulated the protein expression levels of Bax. This is further confirmation that p53 may not be involved in the induction of apoptosis by DCQ in hypoxic DLD-1 cells. Alternatively, apoptosis triggering via Bax/Bcl-2 induction might arise via another pathway.

In addition, DCQ radiosensitization effects were found to be associated with changes in the Cox-2 signaling molecule. The anti-apoptotic Cox-2 is an enzyme that converts arachidonic acid to prostaglandins, and is inducible by various stimuli including interleukin-1, hypoxia, radiation, epidermal growth factor, transforming growth factor-β, tumor necrosis factor-α, and several oncogenes [16]. Recent evidence suggests that Cox-2 inhibition may arrest cells in G_2_/M phase through p53 inactivation. However, in the present study, Cox-2 does not appear to be involved in G_2_/M phase arrest of DLD-1 cells when combination treatments were done under oxia, as p-p53 protein expression levels were induced. Since the modulation of protein expression levels was studied in DLD-1 cells, these results may not be extrapolated to other colon cancer cell lines that showed different features with regard to hypoxic radiosensitization.

Our present data show that pretreatment with DCQ under hypoxic conditions induces cell death in DLD-1 cells probably through the reduction of Cox-2 protein. One mechanism for the pro-apoptotic activity of Cox-2 has been the down-regulation of Bcl-2. Although the precise link between Cox-2 and Bcl-2 has not been elucidated, it is interesting to speculate on the potential role of DCQ in enhancing the sensitivity of hypoxic DLD-1 cells to radiation upon the inhibition of Cox-2 and Bcl-2. More recently, celecoxib, a potent and selective Cox-2 inhibitor, was shown to induce apoptosis in human prostate cancer cells by blocking Akt activation, independent of Bcl-2 signaling [27]. Our results correlate with that of celecoxib, since hypoxic treatment with DCQ inhibited the phosphorylation of the Akt prosurvival gene upon IR exposure. Evidence suggests that the Akt/PKB pathway promotes growth factor-mediated cell survival and inhibits apoptosis via modifying the anti-apoptotic and pro-apoptotic activities of members of the Bcl-2 gene family [16]. Cox-2 may represent a downstream mediator of the Akt/PKB pathways.

## Conclusion

In summary, the data presented here indicate that DCQ could be used as a model radiosensitizer to understand the crosstalk between signaling molecules involved in radiation enhancement. This hypoxic cell radiosensitizer is a potentially useful drug that enhances the response of DLD-1 human colon cancer cells to IR. The radiosensitizing efficacy of DCQ is related to the oxygenation status of the cell and the type of tumor cell. In addition, DCQ seems to generate lethal single stranded DNA breaks upon IR exposure. DCQ radiosensitization effects in DLD-1 cells occur mostly through the enhanced induction of G_2_/M arrest under oxia and apoptosis induction under hypoxia. Apoptosis by DCQ in DLD-1 cells is associated with the inhibition of Cox-2 protein levels and the increase in Bax/Bcl-2 ratio.

## Competing interests

The author(s) declare that they have no competing interests.

## Authors' contributions

WI participated in the design of the study, contributed to data acquisition and analysis and in drafting the paper. FG was involved in revising the manuscript critically for important intellectual content. JH participated in performing the Comet assay. MH provided the compound and critically reviewed the manuscript. HM conceived of the study, and participated in its design and coordination and drafted the manuscript. All authors read and approved the final manuscript.
